# Supplementary figures and images for: An Eigenvalue test for spatial principal component analysis
Source: BMC Bioinformatics. 2017 Dec 16;18:562. doi: 10.1186/s12859-017-1988-y (PMC5732370; doi:10.1186/s12859-017-1988-y)

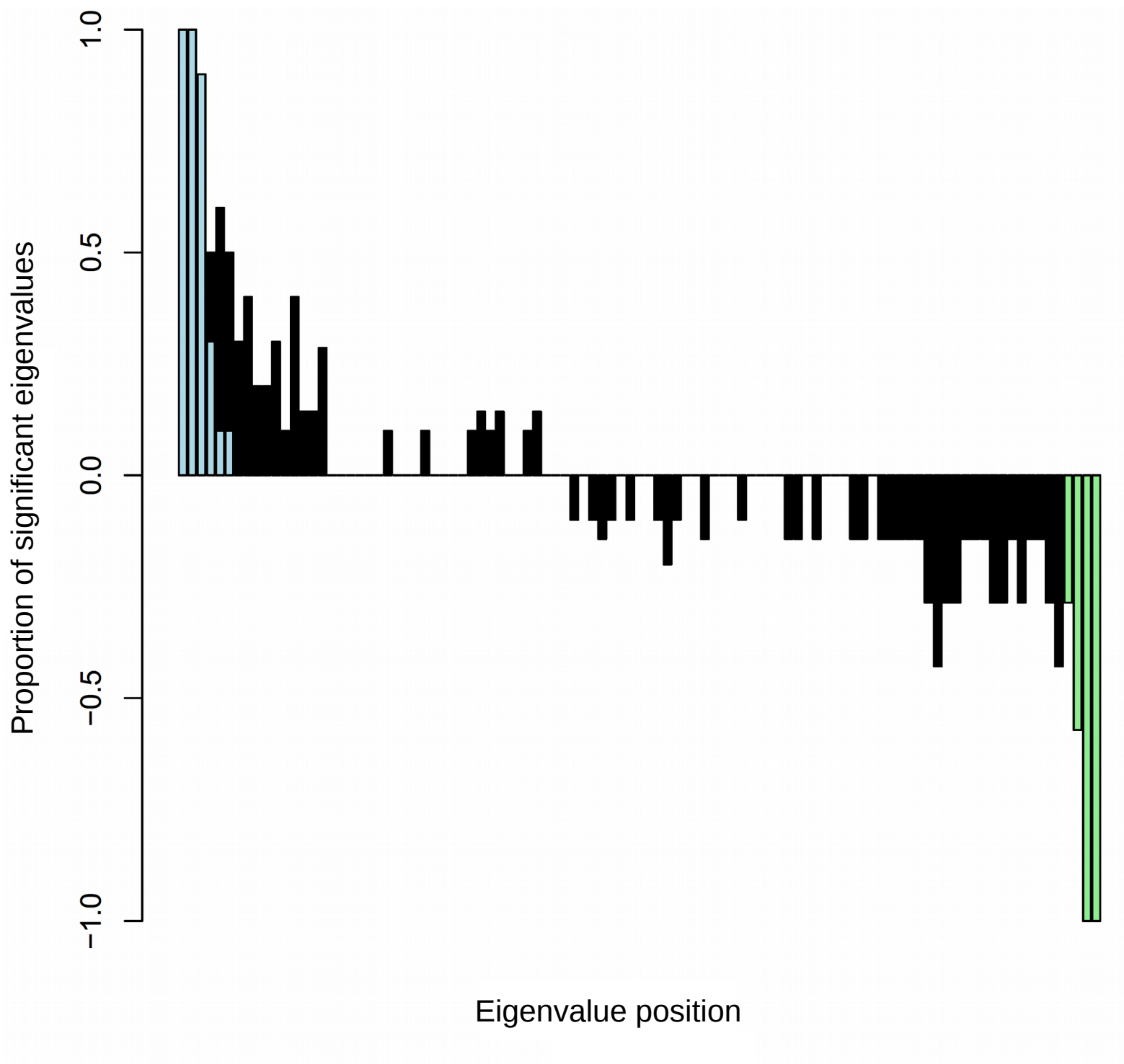

Supplement: Supplementary file 3 — Distributions of significant eigenvalues detected in the presence of global (blue bars) and local (green bars) spatial patterns after hierarchical Bonferroni correction, for 100 significantly positive and 100 significantly negative patterns. Black bars correspond to eigenvalues which are significant without Bonferroni correction. Bars’ height indicates the frequency of observing a significant eigenvalue in a certain position (from most positive to most negative) over the 100 tested patterns. (PDF 1150 kb) [file 12859_2017_1988_MOESM3_ESM.pdf]
